# Modulation of Cell Sialoglycophenotype: A Stylish Mechanism Adopted by *Trypanosoma cruzi* to Ensure Its Persistence in the Infected Host

**DOI:** 10.3389/fmicb.2016.00698

**Published:** 2016-05-11

**Authors:** Leonardo Freire-de-Lima, Leonardo M. da Fonseca, Vanessa A. da Silva, Kelli M. da Costa, Alexandre Morrot, Célio G. Freire-de-Lima, Jose O. Previato, Lucia Mendonça-Previato

**Affiliations:** ^1^Laboratório de Glicobiologia, Instituto de Biofísica Carlos Chagas Filho, Universidade Federal do Rio de JaneiroRio de Janeiro, Brazil; ^2^Instituto de Microbiologia Professor Paulo de Góes, Universidade Federal do Rio de JaneiroRio de Janeiro, Brazil

**Keywords:** *Trypanosoma cruzi*, *trans*-sialidase, immune response, CD8^+^ T cell, glycoconjugates, sialic acid

## Abstract

*Trypanosoma cruzi*, the etiological agent of Chagas disease exhibits multiple mechanisms to guarantee its establishment and persistence in the infected host. It has been well demonstrated that *T. cruzi* is not able to synthesize sialic acids (Sia). To acquire the monosaccharide, the parasite makes use of a multifunctional enzyme called *trans*-sialidase (Tc-TS). Since this enzyme has no analogous in the vertebrate host, it has been used as a target in drug therapy development. Tc-TS preferentially catalyzes the transfer of Sia from the host glycoconjugates to the terminal β-galactopyranosyl residues of mucin-like molecules present on the parasite’s cell surface. Alternatively, the enzyme can sialylate/re-sialylate glycoconjugates expressed on the surface of host cells. Since its discovery, several studies have shown that *T. cruzi* employs the Tc-TS activity to modulate the host cell sialoglycophenotype, thus favoring its perpetuation in the infected vertebrate. In this review, we summarize the dynamic of host/parasite sialoglycophenotype modulation, highlighting its role in the subversion of host immune response in order to promote the establishment of persistent chronic infection.

## The Importance of Sialo-Containing Glycans in Cell Biology

All living cells possess a complex and dense coating composed of glycoconjugates (Varki, 1993). Such structures exist as glycolipids, glycoproteins, and proteoglycans. Among other functions, cell surface glycans mediate cell–cell recognition and control interactions between cells and other components in their local environment. Throughout evolution, glycans were designated several roles based on their physical features and structural diversity (Varki, 2011). Advances in glycobiology researches have provided the means to correlate glycan structures to physiological and pathological conditions (Lowe and Marth, 2003; Baum et al., 2014; Schmaus et al., 2014; Kato and Ishiwa, 2015; Stowell et al., 2015). The result is that glycoscience is emerging as a major contributor to the understanding of biology and medicine. The functional specificity of glycans is often dictated by the monosaccharides at the outermost ends of glycan chains (Berger et al., 1982; Varki, 1993; Hart, 2013). In mammals, the glycan chain of glycoproteins and glycolipids often end with sialic acids (Sia), a group of sugars with distinctive chemical properties that determine their functions (Traving and Schauer, 1998). Discovered about 70 years ago, Sia is a generic term referring to nine-carbon alpha (α)-keto sugars with a carboxyl group linked directly to the ring as well as its *N* or *O* substituted derivatives. There are over 50 Sia described, while the two most commonly found in mammals are *N*-acetyl and *N*-glycolyl neuraminic acid (Varki and Schauer, 2009). It is estimated that a cell exhibits tens of millions Sia molecules, with the concentration in the glycocalyx approaching 100 mM (Varki and Gagneux, 2012). Thanks to its bulky size and the electron density of the carboxyl group, Sia provides an important source of negative charge, which could alter the biophysical properties of cellular interactions. In eukaryotic cells, Sia residues are responsible for mediating many important interactions, including but not limited to regulation of transmembrane receptor function, altering membrane transport, control of glycoproteins (and even cell) half-life (Schauer, 2000). One of the most notable functions of Sia is to regulate immune response, playing an important role in self and non-self recognition (Traving and Schauer, 1998). It should not come as a surprise that several pathogenic microorganisms, such as viruses, bacteria, and protozoa have evolved strategies to exploit this feature (Neu et al., 2011; Nishikawa et al., 2013; Chang and Nizet, 2014). One of the more stylish mechanisms of cell surface sialylation is exploited by the protozoal *Trypanosoma cruzi*, the etiological agent of Chagas disease (Previato et al., 1985; Zingales et al., 1987; Schenkman et al., 1991, 1992; Mucci et al., 2006; Muia et al., 2010).

## *Trypanosoma cruzi trans*-Sialidase and Host Cell Invasion

Incapable of synthetizing Sia, *T. cruzi* has developed an elegant mechanism to take advantage of the Sia residues displayed in its mammalian host sialoglycans in order to avoid detection and elimination by immune cells (Pereira-Chioccola and Schenkman, 1999; Dc-Rubin and Schenkman, 2012; Freire-de-Lima et al., 2015). Since it was shown that *T. cruzi* could not use the activated monosaccharide precursor CMP-Neu5Ac, as it was first considered (Schauer et al., 1983), it was proposed a novel metabolic mechanism of *trans*-glycosylation, by which *T. cruzi* would incorporate Sia onto its own cell surface (Previato et al., 1985). Incorporation of Sia on the surface molecules of *T. cruzi* parasite is catalyzed by the enzyme called *trans*-sialidase (Tc-TS) Previato et al., 1985; Schenkman et al., 1991). It was demonstrated *in vitro* and *in vivo* (Zingales et al., 1987; Previato et al., 1990) the activity of Tc-TS was characterized as specific for α-2,3 Sia, being responsible for the transfer of Sia residues from the mammal host surface sialylated molecules to the parasite mucin-like acceptors (Schenkman et al., 1991; Parodi et al., 1992; Ruiz Rde et al., 1993; Previato et al., 1994).

In *T. cruzi*, the relevance of the acquisition of Sia is not completely known. However, there are proposals that such an event might help trypomastigote forms adhere and penetrate into non-phagocytic cells (Ming et al., 1993; Schenkman et al., 1993). Sia also provides a strong negatively charged cover that protects parasites against human lytic antibodies specific for α-galactosyl residues present on the trypomastigote surface (Pereira-Chioccola et al., 2000).

The transfer of a Sia residue onto an acceptor hinders the access of a second residue when two potential acceptor sites are present on the same oligosaccharide (Previato et al., 1995). Tc-TS can also operate as a viral and bacterial sialidase, irreversibly transferring Sia to water in the absence of a carbohydrate acceptor (Vandekerckhove et al., 1992). Analysis of the crystal structure revealed that the molecular architecture of the Tc-TS active site preserves numerous conserved features of microbial sialidases (Buschiazzo et al., 2002). Furthermore, all sialidases studied so far, including Tc-TS, catalyze sialoside hydrolysis with configuration retention (Todeschini et al., 2000).

There has been significant progress in understanding the importance of Tc-TS on the biology of *T. cruzi*. However, obstacles such as the lack of knockout strains, as well as the absence of specific Tc-TS inhibitors pose difficulties for the progress in the area. In regard to the Tc-TS inhibitors, many of them are not strong enough to serve as a scaffold to the development of drug targeting Tc-TS. In addition, most are derivatives of the substrate Sia or a transition state analog known as 2,3-dehydro-3-deoxy-*N*-acetylneuraminic acid (DANA), with *in vitro* IC(50) values near the milimolar range and low *in vivo* efficacy (Uemura et al., 1992; Amaya et al., 2003; Arioka et al., 2010; Dc-Rubin and Schenkman, 2012). Recent studies suggest that high affinity inhibitory monoclonal antibodies (mAb) for Tc-TS might provide a rational framework for novel approaches in the design of chemotherapeutic drugs (Buschiazzo et al., 2012).

Over the last years, several papers described by Campetella’s group demonstrated that active Tc-TS (aTS) induces systemic effects during the acute phase of the disease (Mucci et al., 2002, 2005; Tribulatti et al., 2005; Risso et al., 2007; Ruiz Díaz et al., 2015), until the elicitation of broad neutralizing antibodies (Pitcovsky et al., 2002). Buschiazzo et al. (2012) reported the identification and detailed characterization of the neutralizing mouse mAb 13G9, which was able to recognize and inhibit Tc-TS with high specificity. The crystal structure of the complex involving the antigen-binding fragment and the globular region of Tc-TS demonstrated that not obstructing the enzyme’s catalytic site, the antibody inhibited the movement of an assisting tyrosine (Y_119_), which plays an important role in the *trans*-glycosidase mechanism (Buschiazzo et al., 2002). The authors suggest that such results may bring into light new strategies for chemotherapy in Chagas disease and for disclosure of aTS function in *T. cruzi* pathogenesis and biology.

Besides encoding aTS, the genes of the TS family also encode its inactive analog (iTS). iTS has a His at position 342, while active members contain a Tyr at the same position (Cremona et al., 1995; Egima et al., 1996). It has been suggested that iTS may mediate the initial contact between *T. cruzi* and host cells, functioning as an adhesin that contains two sugar-binding distinct sites, for Sia and β-galactose (Todeschini et al., 2004). Recently, Ruiz Díaz et al. (2015) demonstrated that both aTS and iTS besides to induce a Th2-like phenotype in naive T cells, also compromise the emergence of Th1 cells. Additionally, both isoforms were associated with the parasite’s ability to reduce IL-2 production and IL-2Ra expression by T cells. The authors suggest that TS proteins are able to manipulate the TCD4^+^ response throughout their maturation stages to favor parasite survival and infection.

The bivalent nature of iTS could promote a glycan cross-linking believed to be essential for cellular signal transduction. Tc-TS has essentially two different domains. The N-terminal, which contains the catalytic domain of the enzyme (Ribeirao et al., 1997), and the C-terminal, which is composed basically of 12 amino acids-long units repeated in tandem, termed SAPA (shed acute-phase antigen; Affranchino et al., 1989), which is not required for TS activity (Campetella et al., 1994). Tc-TS is linked to the parasite membrane via glycosylphosphatidylinositol (GPI) anchor and shed into the bloodstream during the infection (Schenkman et al., 1994). Nowadays, it is well recognized that Tc-TS is an important virulence factor involved in cell invasion and pathogenesis of Chagas disease (Mendonça-Previato et al., 2010; Dc-Rubin and Schenkman, 2012; Miller and Roitberg, 2013; Freire-de-Lima et al., 2015). However, the studies regarding the importance of Tc-TS for cell invasion and parasite survival were paramount to understanding how *T. cruzi* is capable of subverting the immune system by disturbing the host cells sialoglycophenotype.

## Roles of Sialic Acids on T Cell Biology

It is well documented that in T cells, different lectin families recognize distinct sialylated ligands on glycoproteins or glycolipids to regulate their functions (Rabinovich et al., 2002a,b; Ley and Kansas, 2004; Crocker et al., 2007; Crocker and Redelinghuys, 2008). Examples include siglecs, a family of Sia-binding lectins that belong to the superfamily of I-type lectins (Crocker and Redelinghuys, 2008). Most siglecs bind α-2,3 and/or α-2,6-linked Sia on penultimate Gal residues on cell surface glycoproteins or glycolipids; however, some members bind to repeating Sia chains (Varki and Angata, 2006). In the immune system, siglecs mainly function as controllers of cell signaling, although roles for some siglecs in pathogen recognition and innate immunity have been proposed (von Gunten and Bochner, 2008). Erdmann et al. (2009) demonstrated that sialylated ligands on *T. cruzi* interact with Siglec-E (sialic acid-binding Ig-like lectin-E) on dendritic cells (DC), and such interaction suppressed the production of the proinflammatory cytokine IL-12 and subsequent T cell activation. Recently, it was proposed that sulfates from cruzipain, another important antigen expressed by *T. cruzi*, might play an essential role in the interaction with Siglec-E on inflammatory cells, favoring the persistence of the parasite in its mammalian hosts (Ferrero et al., 2016).

Sia plays crucial roles in the regulation of host immunity, as evidenced by the sialylation changes sustained by naïve T cells during thymic selection, a tightly regulated process, essential for establishing central tolerance (Daniels et al., 2001; Moody et al., 2001; Cao and Crocker, 2011; Paulson et al., 2012). The alterations suffered on cell surface *N*- and *O*-linked glycans, including glycoprotein sialylation, during T cell development and differentiation (Daniels et al., 2002) might regulate the T cell response through a direct effect on the intrinsic properties of specific proteins, or by modulating the binding of a disparate set of cell surface proteins to a specific carbohydrate moiety. It is important to bring out that many of the changes in the degree of sialylation of carbohydrate chains observed in T cells during their development can be monitored with plant lectins (Sharon, 1983; Daniels et al., 2002). It has been shown *in vitro* and *in vivo* that T cell activation is accompanied by loss of Sia from core 1 *O*-glycans (Siaα2,3Galβ1,3GalNAc-Ser/Thr; Galvan et al., 1998; Bi and Baum, 2009), which leads to exposure of asialo core 1 *O*-glycans. Such residues can be detected with the plant lectin peanut agglutinin (PNA), that recognizes Galβ1,3GalNAc sequences on several glycoproteins including CD8, CD43, and CD45 (Wu et al., 1996). It is a known fact that the loss of cell-surface Sia during T cell activation enhances TCR reactivity with antigens (Galvan et al., 1998; Harrington et al., 2000; Bi and Baum, 2009). Indeed, T cell cytolytic activity may be boosted by sialidase treatment, with a simultaneous reduction in cell surface negative charge (Sadighi Akha et al., 2006). In addition, de-sialylated CD8^+^ T cells undergo more rounds of cell division following contact with antigen (Pappu and Shrikant, 2004). Recognition and disposal of pathogens rely upon the concerted actions of adaptive and innate immunological mechanisms, including recruitment of different cell types as well as production and release of cytokines, antibodies or other effector molecules (Janeway, 2001). In regards to adaptive response, while the importance of CD8^+^ T cells is well described when it comes to the control of several viral and bacterial infections, it is seldom mentioned that eukaryotic pathogens, such as protozoans, are also subject to the action of cytotoxic lymphocytes. Chagas disease is probably the most well understood example of the role CD8^+^ T cells play during protozoan infections. In its vertebrate hosts, *T. cruzi* is perpetually changing between its intracellular amastigote forms and extracellular trypomastigote ones. There is a great range of cells that are subject to invasion by the parasite, including but not limited to fibroblasts, macrophages, myocytes, and adipocytes. During the intracellular stages, *T. cruzi* antigens are presented by antigen presenting cells (APCs) through by MHC class I molecules, providing many opportunities for detection by CD8^+^ T cells (Garg et al., 1997). Several reports confirmed the importance of CD8^+^ T cell cytotoxicity regarding Chagas disease (Tarleton, 2007, 2015; Padilla et al., 2009; Dos Santos Virgilio et al., 2014). One of the most important was the identification of a number of epitopes expressed in proteins belonging to the TS family genes (Martin et al., 2006). Until that point, there was a lack of immunodominant T cell epitopes, but now it is accepted that members of the TS gene family are a good target for CD8^+^ T cells. That discovery has not only led to a better understanding of how the immune system reacts to *T. cruzi* infection, but in a more practical venue has also fueled the research on vaccine development (Machado et al., 2006; Araujo et al., 2014; Bontempi et al., 2015). Although it is universally accepted that CD8^+^ T cell response is an important factor in mediating host survival during *T. cruzi* infection, there is a substantial delay in the appearance of antigen specific CD8^+^ T cells following infection (Martin et al., 2006; Tzelepis et al., 2006). This delay differs from the quick response of CD8^+^ T cells in other bacterial, viral, and, even protozoal infections (Kaech et al., 2002), suggesting that a mechanism of immune subversion is operative.

## *Trypanosoma cruzi trans*-Sialidase Modulates T Cell Sialoglycophenotype

As mentioned above, Sia content of T cell glycoproteins and glycolipids is regulated during both T cell development in the thymus and activation in the periphery (Moody et al., 2001; Pappu and Shrikant, 2004). Each T cell subset expresses a specific set of glycan-modifying enzymes that regulate the pattern of sialylation on the cell surface (Bi and Baum, 2009). Besides being able to sialylate mucin-like molecules expressed on the parasite surface, Tc-TS is also capable of re-sialylating host cell asialoglycoconjugates (Mucci et al., 2006). Regarding T cells, such event is able to modulate both their function and half-life. Several papers published elsewhere have demonstrated that alterations in Sia residues on thymocytes and mature T cells are able to induce apoptosis (Leguizamon et al., 1999; Mucci et al., 2002, 2005, 2006). Given the way TS works, it comes as a logical conclusion that the parasite’s ability to modulate the T cell sialophenotype is a powerful tool in evading detection and elimination by the host immune system. Pioneering studies on the impact of Tc-TS on T cells (Chuenkova and Pereira, 1995) demonstrated that intravenous injection of minute amounts of native Tc-TS was able to increase the parasitemia and mortality of *T. cruzi*-infected mice. The authors suggested that such effects were entirely dependent on Tc-TS activity, since the same events did not occur in mice primed with viral or bacterial sialidases. Further *in vitro* experiments demonstrated that recombinant Tc-TS binds host T-lymphocytes, activating CD4^+^ T cells through CD43 engagement (Todeschini et al., 2002a,b). However, molecular evidences revealed that CD45 might be the main acceptor of Tc-TS during *T. cruzi* infection (Muia et al., 2010). *In vivo* studies have highlighted the impact of Tc-TS on B and T cells sialoglycophenotypes (Freire-de-Lima et al., 2010; Bermejo et al., 2013), reinforcing the idea that the Tc-TS may act as a virulence factor during *T. cruzi* infection. In B cells, the sialylation of cell surface molecules by Tc-TS elicits the production of IL-17. Interestingly, such event triggers a signaling pathway that differs from the one classically associated with IL-17, bypassing the activation of RORγt and RORα (Bermejo et al., 2013). As for T cells, Freire-de-Lima et al. (2010) demonstrated the relevance of Tc-TS on the sialoglycophenotype of splenic CD8^+^ T cells during *T. cruzi* infection. *T. cruzi*-infected or *Plasmodium berghei*-infected mice were sacrificed on the eighth day post-infection (dpi), and the sialoglycophenotype of CD8^+^ T cells was assessed. As expected, CD8^+^ T cells from *P. berghei*-infected mice were highly positive for PNA (PNA^high^). Interestingly, the glycophenotype PNA intermediate (PNA^int^) exhibited by CD8^+^ T cells derived from *T. cruzi*-infected mice, becomes PNA^low^ after intravenous administration recombinant aTS. Such event was able to inhibit the cytotoxic response mediated by antigen specific CD8^+^ T cells, supporting the idea that re-sialylation of asialoglycans on the surface of activated T cells might be a sophisticated evasion mechanism adopted by *T. cruzi* to subvert the host immune response (Freire-de-Lima et al., 2010). In addition, we found that CD8^+^ T cells from *T. cruzi*-infected mice primed with recombinant iTS exhibited a glycophenotype PNA^high^, suggesting that iTS might have been able to compete with the native aTS by serum sialoglycoproteins, compromising the expected event of re-sialylation that takes place during the acute phase of Chagas disease (Freire-de-Lima et al., 2010). Although the evidence is quite compelling, new experiments with ST3 Gal-I KO mice must be conducted in order to understand the effects triggered by Tc-Ts on CD8^+^ T cells during *T. cruzi* infection. Several immunobiological effects mediated by Tc-TS have been described elsewhere (Mucci et al., 2006; Ruiz Díaz et al., 2015), but the chemical evidence of the enzyme’s essential role in the parasite is still lacking due to the absence of specific inhibitors of Tc-TS. As it stands Tc-TS is a promising target in drug design, an area in which further research is urgently needed.

## Conclusion

Over the last years, several papers have demonstrated the importance of differential sialylation for CD8^+^ T cells with respect to their maturation (Moody et al., 2001; Naito-Matsui et al., 2014), activation (Pappu and Shrikant, 2004), and cytotoxic responses (Sadighi Akha et al., 2006). However, few studies provided insights into the impact of surface sialylation of CD8^+^ T cells on infectious diseases. Infection with *T. cruzi* is of particular interest in this context because the parasite releases into the host plasma large amounts of proteins belonging to the TS family (Frasch, 1994; Schenkman et al., 1994; Giorgi and de Lederkremer, 2011; Freire-de-Lima et al., 2012, 2015). Recent studies demonstrated that cell surface asialoglycans on B and T lymphocytes might be sialylated by the action of Tc-TS (Freire-de-Lima et al., 2010; Bermejo et al., 2013). Such events were able to disturb the host immune response and allow for the continuation of the parasite in the infected host. Since it is universally accepted that Tc-TS acts as a virulence factor during *T. cruzi* infection, further efforts are needed in this lively area to better understand the catalytic mechanism, as well as the functional properties of the Sia-dependent enzyme. Certainly, new impacting findings will contribute to further research on the enzyme as a therapeutic target in the fight against Chagas’ disease.

## Author Contributions

Wrote the paper: LFL, LMF, VAS, KMC, AM, CGFL, JOP and LM-P. All authors read and approved the final version of the manuscript.

## Conflict of Interest Statement

The authors declare that the research was conducted in the absence of any commercial or financial relationships that could be construed as a potential conflict of interest.
